# Modeling the relationship between women’s perceptions and future intention to use institutional maternity care in the Western Highlands of Guatemala

**DOI:** 10.1186/s12978-017-0448-5

**Published:** 2018-01-11

**Authors:** Emily Peca, John Sandberg

**Affiliations:** 1 University Research Co., LLC, 5404 Wisconsin Avenue, Suite 800, Chevy Chase, MD 20815-3594 USA; 2 Milken Institute School of Public Health, Department of Global Health, The George Washington University, 950 New Hampshire Ave. NW, Washington, DC, 20052 USA

**Keywords:** Maternity care, Care-seeking, Quality of care, Mistreatment, Disrespect and abuse, Satisfaction, Childbirth, Guatemala, Indigenous populations, Client perceptions

## Abstract

**Background:**

Despite global gains, women in hard-to-reach areas are at a relatively higher risk of death and disability related to childbirth. Traditional methods of measuring satisfaction may mask negative experiences (such as disrespect and abuse) that can drive down demand for institutional care. Better measurement of women’s perceptions of care quality, especially among marginalized populations with historically low utilization of institutional care, are needed to inform how to improve services and foster greater utilization of (potentially life-saving) clinical care.

**Methods:**

A population-based household survey was conducted in 15 purposively selected villages in the rural Western Highlands of Guatemala among women who gave birth to a child within the last 5 years. Demographic and health information including experiences and perceptions of maternity care were collected. Two sets of nested multivariate logistic regression models were estimated to identify factors associated with future intention to give birth in a health facility, separately among women who gave birth to their last child at home and women who gave birth to their last child in a facility. The independent variables of interest were access to care, perceived need for maternity care, and two measures of perceived quality: satisfaction with last birth and disrespect and abuse (perceived or experienced). Thematic analysis was performed on open-ended responses.

**Results:**

Perceived need for facility-based childbirth services and satisfaction with last childbirth experience, either at home or in a facility, emerged as the key factors influencing intention to give birth in a health institution in the future. Among the facility birth group, reporting disrespect and abuse is a deterrent to seeking facility-based care in the future. However, select perceptions of disrespect and abuse did not have an association with future intention (among the home birth group).

**Conclusions:**

Women’s perceptions of care quality influence care-seeking. Women who feel they were mistreated in health facilities are more likely to avoid or delay seeking care in the future. Health systems need to reinforce trust and positive perceptions of respectful care. Developing better measures of women’s perceptions of maternity care experiences among indigenous populations in Guatemala can inform improvements in care provision.

## Plain English summary

Women in low- and middle-income countries are increasingly giving birth alongside skilled workers in health facilities. However, many women and their families avoid institutional childbirth care leaving them at risk when complications arise. A frequently cited reason for giving birth at home is negative opinions of institutional childbirth care. This study investigates barriers and facilitators to giving birth in a health facility through the use of a household survey conducted among women of childbearing age in a largely indigenous rural population in the Western Highlands of Guatemala. Women who experienced institutional childbirth either at home or in a facility participated. Those surveyed were asked about specific factors thought to be most related to where women intended to give birth including: access to institutional care, whether they experienced or perceived disrespect and abuse was associated with institutional care, satisfaction with last childbirth experience and whether women believe in a need for facility based care. As part of the survey, women were also asked to explain certain answers. The results indicate women’s perceived need for and satisfaction with their last birth experience most influenced intended future delivery location; and experiencing disrespectful care in a health facility was a barrier to future facility birth. Improved methods of capturing women’s experiences and perceptions of care can help us better understand their past choices and, going forward, inform how to increase demand for and improve institutional childbirth services and related programming.

## Background

In 2015, it was estimated that 303,000 women across the globe died due to complications associated with childbirth [1]. Despite average gains, regional estimates mask disparities in health outcomes within countries. Those at highest risk of not receiving adequate care are the geographically isolated, rural poor, residing in certain low- and middle- income countries [2]. Failure to address the needs of “left behind” populations will hinder the achievement of national and global maternal health targets and goals such as universal health coverage (UHC) and the Sustainable Development Goals (SDGs) [3]. Poor outcomes for mothers and babies can largely be prevented through access to emergency obstetric care (EmOC) provided by a skilled birth attendant operating in a sufficiently equipped health facility [4].

To increase use of potentially lifesaving obstetric care we must understand why uptake of services is low. The answer is driven in part by the sociocultural beliefs related to childbirth practices, whether women and families have a perceived need for facility-based care, and if women (and those in their spheres of influence) determine care provided in health facilities is of sufficient or acceptable quality [5]. Despite decades of work invested in measuring experiences and perceptions related to the quality of institutional healthcare, conceptual and methodological issues remain [6]. The challenge has been to construct valid and useful measures of perceptions related to healthcare services, including those specific to the perceived quality of labor and childbirth [7]. Historically, the most common measure of an individual’s healthcare experience is ‘satisfaction.’ Despite popular use, measures of satisfaction have been criticized for a lack of definition and common conceptualization [8]. While it is generally accepted that ‘satisfaction’ represents a collection of distinct interactions and perceptions (which may include a variety of negative and positive experiences), it is often measured as a single-item quantitative measure [7]. Disentangling the confluence of factors that drive satisfaction ratings is a formidable challenge [9].

There are few examples of how to quantitatively measure particular dimensions of poor quality of institutional childbirth care in low- and middle-income countries [10]. One promising approach is the use of the disrespectful and abusive care typology proposed by Bowser and Hill [11]. This typology categorizes specific elements of care (as opposed to the overall experience) by areas identified in the literature as problematic e.g. non-consented care, non-confidential care (including lack of privacy), abandonment/neglect, non-dignified care (e.g. verbal abuse and poor communication), physical or sexual abuse, detention in health facilities for failure to pay (this category has since been expanded to “unfair requests for payment” [12]) and discrimination [13]. In measuring specific dimensions of poor healthcare quality, such as disrespect and abuse, we can hold health systems accountable for what might be masked by (often inflated) measures of satisfaction.

Another (less common) global measure of perceived healthcare quality is ‘willingness to recommend a facility’ to others [14]. ‘Willingness to recommend’ measures have utility in contexts where health service information is passed primarily through social networks. Some argue this is a more accurate measure of satisfaction and indication of future behavioral intentions [15]. Satisfaction and willingness to recommend have been shown to have varying degrees of correlation [14, 15]. Level of satisfaction may not necessarily translate into an equivalent willingness to recommend, suggesting the factors that influence each may be different [14]. Satisfaction measures may tap into an affective evaluation of care [8], while willingness to recommend, could indicate an openness or intention to return to a provider in the future [15]. Therefore, there is a case to be made for evaluating satisfaction and willingness to recommend as separate constructs in their association with institutional childbirth care [14].

The current study explores how women’s first-hand experiences of institutional childbirth care and their perceptions of institutional childbirth care (derived from hearsay, second/third hand accounts of others) are associated with intentions to give birth in a health facility. While intention is an imperfect predictor of behavior, evidence from Bangladesh and Ethiopia suggests intention to give birth in a facility is a significant predictor of whether a woman delivers in a health institution^1^ [16, 17]. The present study draws on quantitative and qualitative community-based survey data from the Western Highlands of Guatemala. This paper is one of the first attempts to model women’s experiences and perceptions of institutional childbirth care on future care-seeking intentions. Assessing women’s opinions of care in the context of other key care-seeking factors, such as individual characteristics and access, can shed light on the relative importance of and associations between them. This analysis can inform how to prioritize interventions to increase uptake of facility-based delivery services in an area with relatively low utilization.

**Fig. 1:**
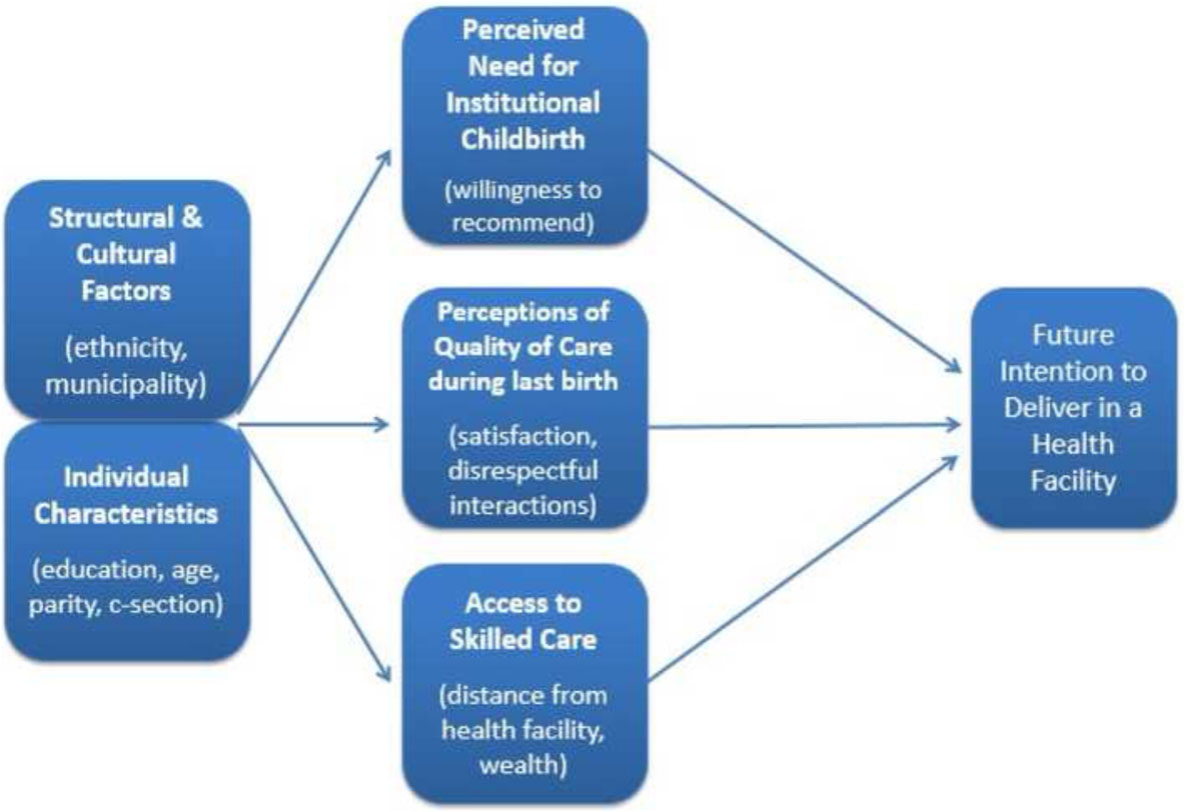
Conceptual model of future intention to deliver in a health facility

The authors developed a conceptual model (post data collection), which illustrates the underlying hypotheses to be tested. The conceptual model shown in Fig. 1 is based on the care-seeking literature and theories of health behavior and health service utilization. Andersen’s model of health service utilization (1960) [5] frames the types of factors hypothesized to facilitate use of health services. These include predisposing characteristics (demographic, social structure and health beliefs), enabling resources (personal/family, community) and perceived need [5]. Building on this frame, the literature identifies key factors associated with where women give birth. These include socio-cultural factors [10, 18, 19]; geographic and economic access [10, 18, 19]; perceived need for facility-based delivery care [10]; previous childbirth experience [18]; and perceived quality of care in health institutions [18, 19]. The conceptual model is concordant with the theory of reasoned action and theory of planned behavior [20], which posit intention to do something is a function of attitudes (behavioral beliefs, evaluation of behavioral outcomes), subjective norms (normative beliefs and motivation to comply) and control over decision-making (control beliefs and perceived power) [21].

Figure 1 illustrates the Conceptual Model of Future Intention to Deliver in a Facility, including the variables from the dataset that are used to operationalize it for the purposes of analysis. Starting on the left-hand side of the figure, intention to use facility-based care is formed by structural and cultural factors (ethnicity, municipality), and individual characteristics (education, age, parity, Cesarean section); these factors shape perceived need for institutional childbirth, perceptions of maternity care quality, and access to skilled care. The conceptual model was developed prior to data analysis and reflects the authors’ hypothesis that the combination of these factors contributes to future intention to deliver in a health facility.

### Setting

Latin America is a region marked by extreme inequity and inequality, which hinders health and human development. In Guatemala, indigenous populations have a history of social and economic marginalization associated with disparities in health and development indicators compared to non-indigenous groups [22]. Three and a half decades of civil war disproportionately devastated indigenous communities in the Western Highlands of Guatemala. Prior to the Peace Accords in 1996, nearly half the entire population had no access to health care [23] until the Government of Guatemala began contracting non-governmental organizations (NGOs) to manage rural health services through the “*Extensión de Cobertura*” or Extension of Coverage Program in 1997 [23]. In addition to increasing access, examples of attempts to address the health needs of indigenous populations include the establishment of the Traditional and Alternative Medicine Program in 2004, the creation of the Unit of Indigenous Populations’ Health Care and Interculturality (2009) [24] and the passage of the *Ley de Maternidad Saludable* or Healthy Motherhood Law in 2010 [25]. The Healthy Motherhood Law includes respect for traditional and cultural practices of indigenous populations and reinforces public services should be free and culturally and geographically accessible —with particular emphasis on marginalized populations.

During the past 15 years, Guatemala has made progress toward decreasing its maternal mortality ratio from 205 per 100,000 in 1990 to 88 per 100,000 in 2015; however, it remains higher than the regional average for Latin America (60 per 100,000) [1]. Additionally, estimates suggest maternal mortality is twice as high among indigenous populations (roughly half of the population) compared to non-indigenous populations (referred to as *ladinos*) [26]. The proportion of women who give birth in health facilities has risen from about 50 to 65% nationally, but ranges from just over 90% in the capital to 36% in the predominantly rural Department of El Quiché, where a high concentration of indigenous populations reside [27]. In El Quiché, 91 % of women report attending antenatal care services provided by a nurse or doctor [27]. For facility-based births, El Quiché (compared to other Departments) has the highest proportion attended by clinically trained midwives (61%), with the remaining assisted by doctors (33%) and nurses (3%) [27]. Language barriers, poor access to services, low literacy and historical marginalization reinforce home birth assisted by *comadronas* (traditional midwives with limited to no clinical training) [28–30]. In Guatemala, the low demand for institutional care (particularly among rural indigenous populations) stems from a lack of perceived need for and acceptability of institutional childbirth care quality [31–34].

El Quiché, the Department^2^ in which the study data were collected, is located in the Western Highlands region and has some of the poorest health and development indicators in Guatemala [27]. El Quiché is largely rural, mountainous and heavily populated by indigenous Mayan groups [29]. The Ixil Health Area, located in the Department of El Quiché, is comprised of three municipalities: Santa Maria Nebaj, San Juan Cotzal, and San Gaspar Chajul. There is one district hospital in Nebaj and two Permanent Health Centers, one in each of the other two municipalities. The Health Centers have the capacity to provide childbirth services (though not Cesarean sections) along with initial management of maternal, neonatal and child complications and referral. (A reliable formal ambulance system did not appear to be present; transport to health facilities relies on pooling resources and gaining access to the nearest vehicle in a village). While there are private facilities in the capital towns within each of the three municipalities, the vast majority of the populations living in the rural areas of Ixil only have access to free government services.

## Methods

Data for the present analysis are drawn from a household survey concerning maternal and child health among women ages 16 to 51 from 15 villages in Ixil. The data were collected in 2014 as part of the Translating Research into Action (TRAction) Project managed by University Research Co., LLC. Study villages were purposively selected to represent a diversity of residences at different proximities to the only public birthing facility in the municipality. Villages ranged from rural (just outside the municipal centers) to remote (roughly an hour plus car ride from municipal centers, including some villages with poor/no vehicle access). None of the study villages were located on the fringes of the municipal centers.^3^ The quantitative and qualitative survey data were collected orally by enumerators in Spanish or Ixil (the predominantly spoken indigenous language) depending on respondent preference. Data collectors were women from Ixil possessing similar cultural and linguistic characteristics as the study respondents, but were not from the study villages. The data collectors were hired by the local NGO named COTONEB (*Cooperativa Todos Somos Nebajenses*), which (at the time) managed health services in Ixil as part of the government’s Extension of Services Program. Data collectors were trained by survey experts contracted by University Research Co., LLC (URC) based in Guatemala. All study participants provided consent before engaging in data collection. Ethical and technical approval were given by URC and the University of San Carlos in Guatemala prior to data collection. URC granted permission to use the data for the present analysis.

Based on census data collected prior to the project, every woman with a child under five (*n* = 754) was eligible to take the maternal and child health survey. Five hundred and eighty-eight women completed the home birth satisfaction questionnaire (roughly 80%) and 153 women completed the facility birth ‘satisfaction’ questionnaire. Eleven individuals refused to participate. Equal proportions of women from the three municipalities participated, but slightly more women from close villages compared to the intermediate and far villages completed the survey. The final analytic sample for the home birth group was 524 and 130 for the facility birth group. The slight decrease in the analytic sample was largely due to missing values resulting from ‘don’t know’ responses to the question concerning future intention to deliver in a health facility.

For the quantitative analysis, two complementary set of models are estimated using data from a home birth group and a facility birth group. The two analyses allow for considering differences in intention among those with recent experience using childbirth services for their last birth (facility birth group) and those who gave birth to their last child at home (home birth group). The home birth group’s intentions to deliver in a health facility in the future may be influenced by experiences with prior births, or their perception of facilty-based care informed by second-hand experience or hearsay, rather than first-hand experience. Since the questions refer exclusively to women’s last births, we we cannot rule out the former. We believe however, based on the overall prevalence of facility birth in the population and insights from a separate qualitative analyses (not shown) that the likelihood of this is low.

The first step was to estimate descriptive statistics (means and proportions) for all variables used in the analysis, followed by an inspection of the bivariate associations between the independent and dependent variables for each of the two analyses. Inferential statistics are presented to illustrate differences when comparing population characteristics and outcomes between the home and the facility group. Then, we estimate multivariate models to detect independent and potentially mediating effects of perceived need, perceived quality, and access on future intention to deliver in a health facility. The multivariate analyses are informed by the conceptual framework and tested in the form of a series of nested logistic regression models. Marginal and discrete change in probabilities are reported.^4^

While inferential statistics are used, these data concern the entire population of women with a child born in the past five years located in the 15 study villages. As such, the estimates presented are population parameters. While inferential statistics are presented in the analyses that follow, these may be best interpreted as approximate measures of the variability around the parameters they represent; from a counterfactual perspective, this population may be thought of as representative of similar (mostly indigenous) populations in the country.

### Quantitative measures

#### Dependent variable

The dependent variable ‘future intention to give birth in a facility’ was captured by the question: “If you were to have more children, where would you like to give birth?” The answer choices were hospital, permanent health center (CAP in Spanish), home, other or “don’t know.” The responses were coded 1 = health facility (hospital/CAP) and 0 = home. The 10 “don’t know” answers from the home group and the six from the facility group along with two “other” responses were not included in the analysis. The same question was asked to the home and facility birth groups.

#### Independent variables

*Health facility access* is operationalized by two variables, proximity to a government health facility equipped to provide childbirth services and household wealth. Proximity to a health facility is coded as 1 = (relatively) proximate, 0 = intermediate/remote. Household wealth is a continuous variable derived from a factor score comprised of 11 dichotomous questions pertaining to household assets and indicators of extreme poverty (factor score items can be made available upon request). Relatively closer proximity to a health facility and greater wealth are hypothesized to increase the probability of intending to deliver in a health facility in the future.

Based on the thematic analysis of the open-ended responses, ‘willingness to recommend a facility to someone else’ (1 = yes, 0 = no, “don’t know” responses^5^ were not included in the analyses) proved to be a solid proxy for *perceived need for institutional childbirth*. (This is explained further in the qualitative results section).

*Perceptions of quality of care during last birth* are measured though questions pertaining to women’s perceptions and experiences of disrespect and abuse and a global satisfaction question. The measures of disrespect and abuse used in both analyses pertain to facility-based childbirth care (since care seeking for institutional services is the phenomenon of interest) but differed by group. Participants in the facility group were asked three questions related to *experienced* disrespect and abuse during their last delivery in a health facility. The three questions included a single-item question that captured ‘any disrespect and abuse’ and two additional items pertaining to non-dignified care and abandonment. The three questions were turned into a composite score in which reporting “yes” to any of the three was coded 1, and 0 meant responding “no” to all three questions. The home group was asked about two *perceptions* of disrespect and abuse related to giving birth in a facility. The first pertained to abandonment: “Do you know or have you heard about women being neglected while utilizing facility-based childbirth services?” The second question was about unfair requests for payment or bribes; participants were asked: “Did you know or have you heard about paying or giving something for better care in health facilities?” Experiencing or perceiving disrespect and abuse in health facilities is hypothesized to decrease the probability of intending to deliver in a health facility in the future. These indicators were chosen because based on the literature they were most relevant in the current context and because the measures were accepted by the local data collection team (some disrespect and abuse questions were viewed as too sensitive and were not included). Detention in health facilities was not seen as prevalent so “unfair requests for payment” was used. All categories of disrespect and abuse are more extensively explored in a qualitative analysis, forthcoming.

The satisfaction question asked to both groups (translated into English) was, “How did you feel about the care you received during your last birth (at home/in a health facility)?” Responses were recorded on a five-point Likert scale. The satisfaction variable was coded 1 = very good/good and 0 = average, bad, and very bad.^6^ The hypothesis is that high satisfaction will be correlated with a future intention to return to the location in which they gave birth last time (e.g. highly satisfied home birth group will intend to give birth at home in the future and highly satisfied facility group will be more likely to intend on giving birth in a facility in the future).

#### Controls

The controls for this analysis are structural and cultural factors and individual characteristics. Proxies for structural and cultural factors included maternal language (1 = indigenous, 0 = Spanish) and respondent’s municipality (1 = Nebaj, 0 = Cotzal and Chajul). Maternal language (also an indication of ethnicity) are expected to have a negative association with facility-based delivery. This is because of indigenous populations’ negative perceptions of institutional care quality based on the predominance of Spanish-speaking staff, who have been known to discriminate against indigenous populations who speak languages besides Spanish [33, 35]. Municipality is included because each of the three municipalities of Ixil has a relatively distinct culture and each has one public facility capable of providing childbirth care; the former may lead to variation in perceptions of quality among the three health facilities. Cotzal and Chajul have relatively new permanent health centers (CAPs) that provide childbirth services, while Nebaj is home to the only public referral hospital in Ixil, capable of managing obstetric complications.

Individual characteristics include indicator variables for education/literacy (1 = yes), number of children at the time of the survey (1 = more than 5 children, i.e. above the departmental average of 5.2),^7^ as well as a continuous measure for respondents’ age. Both high parity and low age may be associated with intention to utilize facility-based care. The thematic analysis suggests *primigravida* women (with prolonged labor) and women of advanced reproductive age are viewed as being higher risk and in possible need of facility-based care. Having at least some education/literacy is hypothesized to increase the probability of intending to give birth in a health facility, through either exposure to information concerning the benefits of institutional care or through a cultural mechanism influencing perceived need. Additionally, the facility group analysis controls for type of last birth (1 = vaginal and 0 = planned/unplanned Cesarean section). This is because having a vaginal versus surgery-assisted birth are different experiences that may impact recommendations and future delivery location preferences.

### Qualitative data

Qualitative data were collected through open-ended responses asked after select survey questions related to the woman’s experience of care during her last childbirth experience. The home birth group was asked to explain their satisfaction scores, and the facility group was asked to explain their affirmative responses to the single-item disrespect and abuse question. Additionally, both groups were asked to give insight into their response to the ‘willingness to recommend a health facility’ question.

The thematic analysis reinforced the conceptual grounding and design of the statistical models, and for the purposes of contextualizing the quantitative results. An inductive thematic analysis was conducted by the lead author (fluent in Spanish) and validated by a team member from the study area who is fluent in both Spanish and Ixil. The open-ended data analysis included a full review of each response (in Spanish) and notation of emergent codes or sub-themes, separately by home and facility group. Then, the sub-themes were grouped according to salient higher order themes and summarized into key findings.

## Results

### Qualitative findings

The data from the 654 open-ended responses (100% response rate) revealed that the ‘willingness to recommend’ construct represents a perceived need for facility-based care. However, this perceived need—and confidence in facility care—appears to largely apply to when obstetric emergencies arise and the belief that facility personnel and infrastructure are best equipped to save lives in these situations. Therefore, willingness to recommend does not appear to constitute a general endorsement of obstetric health service delivery or access. The explanations for why a woman would recommend facility delivery are similar in both the home and facility birth groups. Both groups identify safety and clinical interventions in health facilities as benefits, and distance, cost and quality of care deterrents.

More than half (61%) of the home birth group said they would recommend a facility-based delivery to someone else. Only about a quarter of these women said they intend to give birth in a facility themselves, however. While the majority of the facility birth group feels comfortable recommending a facility birth to someone else, just less than a quarter of these women would intend to give birth in a facility next time. The qualitative data suggests facility births are viewed as a ‘safer’ necessity, but not a preference. One woman summed it up by saying, ‘whether you like it or not, the doctors are the only ones who can save our lives.’ A significant group of women in the home birth group did not feel confident they knew enough about facility-based childbirth services to recommend it to someone else; and some women from both groups were reluctant to share an opinion with others for fear of being teased or scolded if something bad happened because of her recommendation. A substantial portion of the home birth group and some from the facility group indicated they would not recommend a facility birth to someone else because of the poor care provided in health facilities; they often implied care rendered at home was better. Finally, facility birth is viewed as more expensive than home birth and a few women indicated physical access to health facilities is a challenge.

The analysis of the open-ended satisfaction responses among the home birth group (100% supplied a response) underscored an expected preference for home birth. However, the few average to very poor satisfaction ratings were largely accompanied by explanations of disrespect and abuse (largely abandonment/neglect, non-dignified care). The open-ended responses to the overall disrespect and abuse question among the facility birth group (89% supplied a response) validated an understanding of the question as explanations highlighted instances of disrespect and abuse outlined by Bowser and Hill’s typology.

### Descriptive statistics

Table 1 presents the summary of descriptive statistics for the study population. One fifth of the women included in the study reported their last birth was in a health facility. Forty-four percent lived in a proximate versus a distal village. Self-reported ethnicity and maternal language are very highly correlated, with 94% identifying as indigenous (compared to *ladina*), but slightly fewer (84%) reported an indigenous maternal language (majority Ixil, some K’iche, and minority Kanjobal). Nearly half of the study population was literate or had completed some education. At the time of the survey, the ages of the participants were 16 to 51 years with a mean of 28 years. The women reported the number of their offspring at the time of the survey, which ranged from one to 15; one-fifth of the women reported six or more children. Only 4 % of the facility group delivered their last child via Cesarean section with the vast majority reporting a vaginal birth.

**Table 1:** Characteristics of the home and facility groups

Variable	Home group	Facility group
	*N* = 524	*N* = 130
	mean/proportion (standard deviation)	mean/proportion (standard deviation)
Determinants of intention, facility birth		
Distance	40%	62%**
1 = proximate, 0 = distal	(0.491)	(0.488)
Municipality	37%**	25%
1 = Nebaj, 0 = Cotzal/ Chajul	(0.483)	(0.437)
Maternal Language	87%	70%**
1 = indigenous, 0 = Spanish	(0.332)	(0.460)
Wealth Factor Score	−0.022	0.096
0.002; range: −.984-3.624	(0.718)	(0.764)
Parity	21%	16%
1= > 5 children	(0.405)	(0.369)
Education	46%	54%*
1 = some, 0 = none	(0.499)	(0.500)
Age	28.8	28.1
16-51	(7.479)	(7.691)
Birth Type	n/a	80%
1 = vaginal, 0 = C-section	(0.076)	(0.402)
Independent Variables		
Willingness to recommend a facility birth to others	55%	85%**
1 = yes	(0.498)	(0.355)
Satisfaction with care during last delivery	92%	91%
1 = Very good/good 0 = average to very poor	(0.278)	(0.291)
Experienced D&A	n/a	18%
1 = yes to at least one of the three D&A questions		(0. 383)
Perceptions of abandonment in health facilities	10%	n/a
1 = yes	(0.294)	
Perceptions of “unfair requests for payment” in health facilities	9%	n/a
1 = yes	(0.280)	
Outcome		
Intends to deliver next child in a health facility	16%	72%**
1 = yes	(0.369)	(0.453)

Source: Compiled by author using COTONEB survey data

Statistical difference between groups denoted by *p < .10, **p < .05

When comparing the characteristics and responses of the two groups as illustrated in Table 1, there are statistically significant differences by location, maternal language and education. Significantly more facility group women are from proximate villages within the municipalities of Cotzal and Chajul relative to Nebaj. The facility group also has a significantly higher proportion of educated/literate women and those who speak Spanish as a maternal language compared to the home birth group. There are no significant differences between groups in terms of wealth, parity and age.

The overwhelming majority of women from both groups (greater than 90%) reported the two highest ratings of satisfaction related to the care they received during the birth of their last child. The satisfaction responses were similarly distributed across the five categories for both groups of women. As for the quality variables, 18% of the facility group reported they *experienced* at least one of the three disrespect and abuse scenarios comprising the composite score. Fifteen percent of the home group indicated they *perceived* or believed at least one of the two examples of disrespect and abuse (abandonment or unfair requests for payment) are present during facility birth.

In the overall study population, 61% would recommend a facility-based delivery to someone else and 27% intend to deliver in a facility next time. Significantly more women from the facility group reported willingness to recommend and a future intention to return to a health facility to give birth compared to the home group. Just half of the home birth group would recommend a facility-based delivery, and only 16% intend to give birth to their next child in a health institution.

### Bivariate results

Table 2 displays the bivariate estimates of future intent to deliver in a health facility and the independent variables of interest (access, perceived quality, and perceived need). Starting with the home group and access variables, wealth is negatively associated with future intention to give birth in a health facility. For distance, there is a statistically significant relationship with the outcome suggesting that the discrete probability of intending to deliver in a health facility among those living in a relatively proximal village is 0.11 higher compared to those from more distal villages. The association between the two perceptions of disrespect and abuse (abandonment and unfair requests) with future intention to deliver in a health facility is positive in the home group. The probability of intending to deliver in a health facility in the future is 0.19 lower for those who were satisfied with their last birth at home compared to those who were dissatisfied with their last birth.

**Table 2:** Marginal/Discrete changes in the probability of future intent to deliver in a health facility

Independent variable	Home group	Facility group
	*N* = 524	*N* = 130
	(SE)	(SE)
Perceived quality		
Perceptions of abandonment in health facilities	0.039	n/a
1 = yes	(0.051)	
Perceptions of “unfair requests for payment” in health facilities	0.112**	n/a
1 = yes	(0.047)	
Experienced disrespect & abuse		−0.166*
1 = yes to at least one of the 3 D&A questions	n/a	(0.082)
Satisfaction with care during last delivery	−0.190**	0.132
1 = Very good/good 0 = average to very poor	(0.044)	(0.126)
Perceived Need		
Willingness to recommend facility birth to others	0.215**	0.467**
1 = yes	(0.029)	(0.119)
Access		
Wealth	−0.017	0.049
	(0.024)	(0.049)
Distance	0.111***	−0.008
1 = proximate, 0 = distal	(0.030)	(0.081)

Source: Compiled by author using COTONEB survey data

Note: *p < 0.10, **p < 0.05, ***p < 0.010

Turning to the facility group, distance has a very weak negative association with the outcome, which is contrary to the hypothesis that closer proximity increases the probability of intending to deliver in a health facility. The association between experienced disrespect and abuse and intention to have an institutional birth is negative among the facility group. Specifically, the estimated probability of intending to repeat a facility birth among women who experienced disrespect and abuse is estimated to be 0.16 lower compared to those who did not. Further, high satisfaction with last birth in a health facility is associated with a higher probability (0.13) of returning to a health facility to give birth in the future.

The association between willingness to recommend and future intention to deliver in a facility is positive and statistically significant for both groups. The estimated difference in the probability of intending to use a facility in the future between those willing to recommend versus those unwilling to recommend is double the magnitude in the facility group compared to the home group.

### Multivariate results

#### Future intention to give birth in a health facility – home group

Table 3 displays the multivariate results related to future intention to deliver in a health facility among the home group participants. Model 1 includes the control variables represented by structural and cultural factors, as well as individual characteristics (language, ethnicity, age, literacy and parity). The estimates indicate, on average, speaking an indigenous maternal language, living in the municipality of Nebaj, and an above-average number of children are negatively associated with future intention to give birth in a health facility. Further, age and having some education/literacy are positively associated with intending to have an institutional birth in the future.

**Table 3:** Changes in probability of reporting future intent to deliver in a health facility (home birth group only) *N* = 524

Variable	Model 1	Model 2	Model 3	Model 4	Model 5
	(Determinants of intention/controls)	(Access to institutional care)	(Perceived need)	(Satisfaction)	(Disrespect & abuse)
Municipality 1 = Nebaj, 0 = Cotzal/ Chajul	−0.149***	−0.145***	−0.126***	−0.135***	−0.138***
Language 1 = indigenous, 0 = Spanish	−0.081	−0.089*	−0.076	−0.070	−0.062
Parity 1 = 6-15, 0 < 6	−0.024	−0.014	−0.018	−0.012	−0.019
Education 1 = some, 0 = none	0.078**	0.077**	0.054**	0.052**	0.052**
Age	0.005*	0.004	0.002	0.002	0.002
Distance 1 = proximate, 0 = distal		0.099***	0.081***	0.072***	0.065***
Wealth		0.008	−0.006	−0.006	−0.004
Willingness to recommend facility birth to others 1 = yes			0.188***	0.172***	0.171***
Satisfaction with care during last birth 1 = very good/good, 0 = average to very poor				−0.310***	−0.304***
Perceptions of abandonment in health facilities 1 = yes					−0.008
Perceptions of “unfair requests for payment” in health facilities 1 = yes					0.073
Df	5	7	8	9	11
Model Diff Chi^2^		12.71***	53.75***	24.31***	2.42
Models compared		(1&2)	(2&3)	(3&4)	(4&5)

Source: Compiled by author using COTONEB survey data

*p < 0.10, **p < 0.05, ***p < 0.010

Model 2 builds on the specification from Model 1 to include two facility access variables: distance and wealth. The inclusion of the controls with the access variables adjusts the bivariate wealth estimate, now resulting in a slight positive association with the outcome. The distance (to nearest health facility) association remains statistically significant, adjusted slightly downward compared to the bivariate estimate. The results indicate the discrete probability of intending to deliver in a health facility is 0.99 higher for women living in proximal villages compared to women in distal villages. The results of Model 2 indicate the addition of the access variables improves model fit as evidenced by the likelihood ratio test.

Model 3 adds perceived need for institutional birth, represented by ‘willingness to recommend’ to the multivariate model. As anticipated, the discrete probability of intending to give birth in a health facility for those willing to recommend institutional childbirth is 0.188 higher compared to those who are not willing to recommend facility-based childbirth. The estimated discrete change in the probability of future intention to use a facility associated with willingness to recommend decreases only slightly in the multivariate context compared to the bivariate estimate. The decreased magnitudes of the controls and access estimates imply that perceived need (willingness to recommend) partially mediates the relationship between the controls and access variables and the outcome, future intention to deliver in a health facility.

Model 4 adds the satisfaction variable to the specification from Model 3, capturing the woman’s perceived quality of care during her last birth at home. Compared to the bivariate estimate, the discrete change in probability associated with satisfaction increases by 0.12 in the presence of the controls, access and perceived need variables. This indicates the probability of intending to give birth in a health institution among those who reported high satisfaction with their last home birth is 0.31 lower compared to the women who reported low satisfaction. Minor changes in the control estimates suggest some of their explanatory power is associated with differential satisfaction. Willingness to recommend is partially explained by satisfaction, but not completely, which supports the assumption that the two variables capture different constructs and should be controlled for separately. Model fit is improved by the inclusion of ‘willingness to recommend’ as indicated by the likelihood ratio test (*p* < .001).

Lastly, Model 5 incorporates the two specific negative *perceptions* of facility care quality in the form of unfair requests for payment and abandonment (i.e. two examples of disrespect and abuse). These perceptions of quality are not strongly associated with future intention to deliver in a health facility net of satisfaction, willingness to recommend a facility birth and the controls. The inclusion of the two disrespect and abuse variables have little to no effect on the other covariates and does not improve model fit. Specifically, perception of abandonment in health facilities in the multivariate context (compared to the zero order effect) now has a negative association as expected. The estimate has a magnitude of nearly zero and no substantive meaning, likely due to the inclusion of municipality (analysis not shown). The estimate associated with the measure of unfair requests for payment remains positive and appears to be adjusted by the inclusion of distance (analysis not shown). The two perceptions of disrespect and abuse used in Model 5 do not mediate or explain the associations between willingness to recommend or satisfaction and intent to deliver in a facility.

#### Future intention to give birth in a health facility – facility group

Table 4 below shows the results of a similar set of nested models using the facility group data. This analysis included control variables represented by structural and cultural factors and individual characteristics, similar to the home group analysis. The only difference is the inclusion of type of delivery (vaginal/Cesarean section), which was not relevant to the home birth group. When testing control variables in the context of each other, municipality of Nebaj and indigenous maternal language are significantly associated with a decreased likelihood of intending to give birth in a facility. Specifically, women from the municipality of Nebaj (compared to Chajul or Cotzal) are estimated to have a 0.27 lower probability of intending to return to a facility to give birth. Further, future intention to give birth in a facility is associated with an estimated 0.25 lower probability among those who have an indigenous maternal language compared to women who indicate Spanish is their native language. The direction of association for language aligns with the home group analysis, but has a higher magnitude in this group. Vaginal birth (compared to Cesarean section) and high parity (compared to low) also have negative associations with the outcome. Conversely, increasing age has a positive association with the outcome, as well as education; both estimates have one-tailed tests significant at the 0.1 alpha level.

**Table 4:** Changes in probability of reporting future intent to deliver in a health facility (facility group only) *N* = 130

Variable	Model 1	Model 2	Model 3	Model 4	Model 5
	(Determinants of intention/controls)	(Access to institutional care)	(Perceived need)	(Satisfaction)	(Disrespect & abuse)
Municipality 1 = Nebaj, 0 = Cotzal/ Chajul	−0.270**	−0.262**	−0.334***	−0.324**	−0.312**
Language 1 = indigenous, 0 = Spanish	−0.248***	−0.254***	−0.221***	−0.225***	−0.231***
Parity 1 = (6-15), 0 = (0-5)	−0.117	−0.105	−0.060	−0.035	−0.044
Education 1 = some, 0 = none	0.152	0.158	0.099	0.106	0.108
Age	0.012	0.011	0.006	0.007	0.008
Birth Type 1 = vaginal, 0 = C-section	−0.036	−0.039	−0.075	−0.069	−0.078
Distance 1 = proximate, 0 = distal		0.040	0.051	0.048	0.051
Wealth		−0.014	−0.002	−0.002	−0.001
Willingness to recommend a facility birth to others 1 = yes, 0 = no			0.557***	0.559***	0.548***
Satisfaction w/last birth 1 = very good/good, 0 = average to very poor				0.140	0.097
Experienced D&A 1 = yes to at least one of the 3 items					−0.107
Df	6	8	9	10	11
Model Diff Chi^2^		0.29	18.50***	0.88	0.83
Models compared		(1&2)	(2&3)	(3&4)	(4&5)

Source: Compiled by author using COTONEB survey data

*p < 0.10, **p < 0.05, ***p < 0.010

In Model 2, the access variables (distance and wealth) are added to the specification in the previous model. In the multivariate context, the signs on the access variables change direction, suggesting the bivariate estimates may be distorted. While living in a more proximal village now has a positive association (as expected), increasing wealth has a negative (and low) marginal probability associated with intention to deliver in an institution (a similar result to that seen in the home group analysis). The inclusion of both access variables does not result in an improved model fit and has little impact on the estimates of the controls.

Perceived need (operationalized as ‘willingness to recommend’) is introduced in Model 3. In the multivariate context, the estimate for ‘willingness to recommend’ increases in magnitude compared to the bivariate estimate. Differential perceptions of need for institutional care were previously hidden within categories of municipality, birth type and distance. Controlling for this variation within categories of the indicated covariates increases the overall magnitude of the estimated association between ‘willingness to recommend’ and future intention to deliver in a facility. Decreases in the other controls and access variables suggest perception of need partially mediates the relationship between the former and future intentions to deliver in a health facility.

The probability of intending to return to a health facility to give birth is 0.56 higher among women who reported a ‘willingness to recommend’ compared to the women who were not willing to recommend institutional childbirth. The addition of ‘willingness to recommend’ improves model fit. This proxy for perceived need suppresses the effect of municipality, along with vaginal birth, which nearly doubles in magnitude. The remaining covariates are mildly adjusted downward. Perceived need is suppressed by municipality and type of birth—two factors that theoretically could counteract perceived need for facility-based care.

Model 4 incorporates satisfaction with care provided during last birth in the health facility. Higher satisfaction ratings are still positively associated with future intention to deliver in a health facility. However, the estimate is largely the same in the bivariate analysis and the likelihood ratio test indicates satisfaction does not improve model fit. Satisfaction remains independent of the other covariates, including the other variables hypothesized to mediate access and perceived need.

The final model includes the composite disrespect and abuse variable, which maintains its negative association with future intention to return to a health facility to give birth, as anticipated. Compared to the bivariate estimate, the effect of disrespect and abuse is partially explained by the controls, satisfaction, and most importantly, perceived need (willingness to recommend) (analysis not shown). Again, it appears this second measure of quality (experienced disrespect and abuse) is no longer statistically significant in the context of the covariates, and the estimate indicates it is independent of the controls and access variables (similar to satisfaction).

## Discussion

Across both the home and facility birth groups, the results suggest the care seeking factors of interest—access, perceived need and perceived quality—demonstrate generally expected associations with future intention to deliver in a health facility, with a few exceptions. In terms of access, living in relatively closer proximity to the health facility increases the probability of intending to give birth among the home birth group, but the association was less compelling for the facility birth group. Perceived need, represented by willingness to recommend, was associated with a higher probability of intending to have a future facility-based birth among both groups. For women’s perceptions of care quality, satisfaction with last childbirth experience reinforced intention to return to the same location; and as expected, women who reported experiences of disrespect and abuse during their last facility based delivery had a lower probability of intending to return to a facility in the future. The measures of perceived disrespect and abuse (abandonment and unfair requests for payment) among the home birth group did not demonstrate conclusive associations in this context.

The results indicate the original conceptual model could be modified for each group to better capture the estimated relationships among the variables in this context. For example, the analysis presented here suggests that, in this particular population, the association between cultural, structural, and individual characteristics and future intentions to use facility based maternity care are partially mediated by perceived need for institutional care (in both groups) and satisfaction with prior childbirth experience in the home group. For the facility group, physical access to care and perceived care quality (disrespect and abuse and satisfaction) additionally had independent effects on future intentions to use facility-based maternity care.

The two types of perceived quality of care measures—disrespect and abuse and satisfaction—generated mixed results. For the home group, satisfaction with home birth is the main driver of intentions to give birth at home again. The two measures of perceptions of disrespect and abuse did little to help explain the home group’s future intentions. As originally hypothesized, the facility group analysis indicates poor satisfaction ratings and reports of disrespect and abuse are a deterrent to seeking facility-based care in the future. Further, these results suggest satisfaction and the disrespect and abuse measures capture different aspects of perceived quality of care. This is evidenced by the fact that when both measures are estimated simultaneously, satisfaction is adjusted downward in the presence of disrespect and abuse.

The findings from this analysis may be generalizable to other rural indigenous populations in Guatemala, but several limitations must be kept in mind. While a limited number of variables were included, (due to the relatively small sample size,) key factors identified in the literature as being associated with uptake of facility-based childbirth care informed the conceptual model and are included in the models. Other important care-seeking factors may have been omitted from the current analyses, and some measures (structural and social factors and individual characteristics) may be imperfect proxies of these constructs. Going forward it would be useful to include additional information about the facility group care experiences such as duration of the stay in the health facility, cost of care, degree of obstetric complication and health outcomes of mother and baby. These are factors that may negatively influence future intention to seek facility-based care in the future [33]. Additionally, controlling for whether women’s preferences were accommodated (i.e. presence or absence of respectful practices), such as being allowed to give birth in one’s preferred position and observe cultural practices as outlined in the Healthy Motherhood Law [25], may increase future intention to return to a health facility to give birth. For the home birth group, it could be useful to control for religion or spiritual beliefs/values [36] and measures of empowerment or autonomy (given the low status of women in Guatemalan society) [33] as those factors may influence a woman’s ability or intention to use institutional care.

Another limitation may stem from recall bias when questioning women about experiences that may have occurred within a five-year time-frame. However, such biases may be minimal. A study from Ghana suggests that women’s recollections of obstetric events were found to be accurate, even more than five years later, though this may pertain to more tangible events rather than subjective feelings [37]. There is potential uncontrolled for heterogeneity within the home birth group given the possibility that some women may have had a facility birth experience prior to her last home birth, though the assumption is that this was rare and the subsequent effect on the results unsubstantial. The normalization of disrespect and abuse combined with low expectations and relatively less experience with institutional childbirth care may have contributed to “under-reporting” of disrespect and abuse. This should be partially adjusted by the inclusion of education/literacy and wealth; additionally, we are concerned with subjective reports of women’s experiences as they are what drive care-seeking (as opposed to “objective” measures of disrespect and abuse or satisfaction). Courtesy bias may have affected responses to the questions about willingness to recommend or intention to use maternity care in the future. However, the variation and distribution of responses increase confidence in the findings. For example, only 25% would recommend facility birth *and* intend to deliver in a facility in the future, and 37% of the total study population would neither recommend nor intend the former. While there is potential for reverse causality of the association between willingness to recommend and future intention to deliver in a health facility, the analysis of the accompanying qualitative data reveals willingness to recommend is a proxy for perceived need for institutional care. A woman is not likely to report an intention to do something for which she does not perceive a need.^8^

This research contributes to the literature by testing two types of self-reported quality of care measures: a ‘global’ measure of satisfaction and specific measures related to poor perceptions of quality that capture disrespectful and abusive service provision. While there is conceptual overlap between satisfaction with facility care and reporting specific instances of disrespect and abuse, the facility group analysis suggests the measures largely capture different aspects of the relationship between perceptions of quality and future delivery location preference. Global satisfaction ratings often lack variation and are skewed to the positive end of the spectrum [38]. This appears to be true in this case as more than 90% of women reported high to very high satisfaction with their last birth experience at home or in a health facility.

Reported high satisfaction with facility care is positively associated with intent to give birth in a health facility again in the future. However, the facility group’s high satisfaction ratings may be less of an indication of stellar service provision and more a function of low expectations or other confounders [39]. The qualitative data suggest the expectation in the case of a facility-delivery is tied to survival, instead of the traditional expectations of childbirth care in the home. Reports of high satisfaction among the facility group may be a better measure of ‘acceptable’ care instead of ‘highly satisfactory’ care [8]. High satisfaction among the home group was the most substantively and statistically significant factor associated with future birth location. Satisfaction with home care may represent the overall preference for home birth, which is why it is a major deterrent to future intent to seek institutional childbirth care. An analysis of the qualitative open-ended responses reinforces why women prefer to give birth at home.^9^ As hypothesized, satisfaction with (or preference for) home birth was found to mediate perceived need for institutional care, distance and municipality. Future research could further examine the relationship among specific instances of disrespect and abuse experienced during home birth (which was likely partially captured by home satisfaction ratings) and intention to seek facility birth care.

The nested model results from the facility group indicate the satisfaction estimate is adjusted by the inclusion of disrespect and abuse, meaning disrespect and abuse explains variation apart from that explained by satisfaction. Further, only 9 % of the facility group reported average to very poor satisfaction with their care compared to the 18% who reported disrespect and abuse. This supports the hypothesis that satisfaction may represent an affective evaluation of quality that could discount or fail to capture specific (negative) experiences [40]. The results indicate the measures of perceived quality (satisfaction versus experiences of disrespect and abuse), when operationalized in this context, are interpreted as different constructs likely limiting issues of potential endogeneity due to a correlation between the perceived quality measures and the outcome.

The perceptions of disrespect and abuse incorporated in the home group analyses were not significant or substantively associated with future intentions to use facility-based childbirth care. Perceived abandonment in health facilities had the anticipated negative association, but a virutally null estimate. The isolating effects of abandonment were potentially captured by the language variable and municipality of Nebaj where poor care is most commonly perceived. Perceptions that one must pay or give something for better care (unfair requests for payment) is associated with higher likelihood of facility-based delivery intentions. This could be due to the general expectation of costs related to facility-based care. “Unfair requests” may not be viewed as distinct from other payments. Future research should explore other potentially important perceptions of quality that may explain contributors to low intended use of facility-based childbirth care.

The decision about where to give birth is a complex social process that can lead to delays in seeking emergency obstetric care. The qualitative data suggest respondents must balance sub-optimal quality of services with believing health facilities are safer, which is concurrent with other research indicating acceptability of facility birth in the event of an obstetric complication [19]. The qualitative and quantitative responses suggest perceived need is an important mediating factor for both groups’ future care-seeking. Willingness to recommend or perceived need for institutional care demonstrates an awareness of the susceptibility to and severity of obstetric complications, making them more likely to utilize facility-based care [17]. Sixty one percent of the respondents, from both groups, are willing to recommend a facility, suggesting an acceptance of and perceived need for institutional care. However, less than a third of women intend to have a facility-based delivery. This figure is not much higher than the proportion of women from the Department of Quiché who reported a facility birth during the last maternal and child health survey [27].

Among the structural, cultural and individual characteristics, only literacy/some education was seen to be associated with a higher likelihood of intending to deliver in a health institution (for both groups). It is not surprising that the estimated probability of intending to give birth in a health facility for those who are literate/have some education is twice as large for the facility group compared to the home group. Among the facility group, indigenous language was a significant deterrent to future intentions to repeat a facility delivery, indicating indigenous populations may not feel comfortable with the institutional childbirth processes and interactions—potentially a result of their inability to communicate [28] or because of differential treatment of indigenous populations [30]. Women with high parity from both groups have a relatively weak lower discrete probability of intending to deliver in a health facility in the future compared to those with fewer children. This could be a function of having sufficient success at home that one does not perceive a need for facility-based care or lack of intention to have more children. Living in the municipality of Nebaj is also strikingly associated with decreased intentions (overall) to deliver in a health facility, especially among the facility group. This could be because Nebaj’s referral hospital is associated with poorer treatment and/or infrastructure (suggested in a separate analysis of qualitative data, not shown). Alternatively, this health facility offers more surgical and other medical interventions that may be feared by respondents. The potential stigma surrounding the Hospital of Nebaj is unfortunate as this is the only public health facility capable of managing a complicated delivery in Ixil. Age was not significantly associated with future childbirth intentions for either group.

For hard-to-reach communities, access played less of a role in determining future intention than anticipated. Living in closer proximity to health facilities was associated with higher discrete probability of intending to have a facility birth for both groups. Proximity to the health facility was a stronger and more significant predictor of childbirth intentions for the home group compared to the health facility group. Closer access may be a more significant pull factor for the home group because better physical access means less of a perceived investment in time and money to reach care, making the thought of facility-based care utilization more likely. While closer physical access to health facilities does increase likelihood of use, the facility group already may have navigated physical and geographic access barriers to utilize facility care in the past, potentially minimizing the importance of proximity to care when considering future intentions. Notable is the relatively small magnitude of association between wealth and future delivery intentions for both groups. This could be explained by the fact that facility delivery is not a preference, but driven by obstetric emergencies, in which case families find a way to invest in facility-based delivery. This interpretation is reinforced by the strength of the magnitude and direction of ‘willingness to recommend,’ which again, points to the perception of need driving intention to use facility-based care. The findings suggest the access variables may be better categorized as controls since wealth and distance do not appear to play a mediating role as originally hypothesized.

## Conclusions

Women’s perceptions of care quality influence future care-seeking for childbirth services. Facility group women had a lower probability of returning to a health facility if they were dissatisfied with their last care experience or reported being disrespected. Further, the home birth group’s satisfaction with home childbirth care is the driving force for maintaining a preference for giving birth at home. Interestingly, the qualitative data revealed the majority of dissatisfied women who gave birth at home indicated they were mistreated. Whether mistreatment at home could be a driver of facility delivery is an area for future investigation.

This study illustrates that measurement of a complex phenomenon like perceived quality (e.g. examples of disrespect and abuse) can be successfully conducted in geographically hard-to-reach places and executed in multiple languages (e.g. Spanish, Ixil) at a relatively low cost and in a short time period. The willingness to recommend a facility birth question could serve as an indicator of increasing demand in the event of an obstetric emergency and may foreshadow a shift in preferences. This is an example of another indicator that could be further tested more widely in this and other contexts. The use of qualitative data validated the understanding of the quantitative willingness to recommend survey question along with the overarching disrespect and abuse question. There is potential to adapt or build upon the willingness to recommend and disrespect and abuse indicators for future population-based or more routine data collection efforts. These measures could provide a source of data to monitor the implementation of laws, policies and programs; for example, the operationalization of elements related to mistreatment or respectful care programming and practice outlined by the Healthy Motherhood Law and other existing intercultural care guidelines in Guatemala. At the health facility-level, screening for past dissatisfaction and negative experiences as part of the antenatal history to address demand issues could be incorporated as a strategy for increasing facility care. Feedback from clients and continued measurement of specific negative experiences (instead of just satisfaction) can inform efforts required to increase uptake and quality of care.

It is unlikely that the prevailing preference in historically marginalized rural communities will rapidly shift from home birth to facility birth. While utilization is still relatively low, it is promising to see over half of the women from the 15 rural communities signal the acceptance of facility care in the case of an obstetric emergency. The key implication of this work is that a failure to address perceptions of quality and how women are treated in health facilities can ultimately result in a delay or avoidance of seeking institutional childbirth care the future—thus rendering women and newborns more vulnerable to mortality and morbidity related to childbirth. Now is the time to enhance efforts to measure women’s experiences with health facilities and to focus on accountable implementation of policies and guidelines related to advancing respectful care.

## Abbreviations

CAPs: Permanent health centers
COTONEB: Cooperativa Todos Somos Nebajenses
D&A: Disrespect and abuse
EmOC: Emergency obstetric care
SDGs: Sustainable Development Goals
UHC: Universal health coverage
URC: University Research Co., LLC

## Footnotes

1 A study conducted in Bangladesh found the odds of giving birth in a health facility among women who intended to give birth in a health facility were 14.4 times higher compared to women who did not intend to give birth in a health facility [15]. Further, a study from Ethiopia asked women where they intended to give birth and found at follow-up post-delivery that 80% who intended to have an institutional birth actually did [16].

2 Guatemala is divided into 22 departments (similar to provinces) and 334 municipalities (similar to counties).

3 Proximity of villages (e.g. proximate, intermediate, and remote) were determined by the local study team based on their experience traveling to villages within the three municipalities. In very rough terms, the proximate villages were about 10-15 min vehicle ride from the municipal centers, the intermediate villages about 30 min and the remote villages were up to an hour plus ride by car with some villages lacking vehicle access (and in one case electricity).

4 Estimates of marginal (in the case of continuous covariates) and discrete (in the case of categorical covariates) change in probability of an outcome provide a more precise and intuitive way to interpret marginal effects in non-linear models such as those presented here than either model coefficients or odds-ratios. Parameter estimates from non-linear models are not in the metric of the original dependent variable and interpretation is difficult. Transformation of estimates to odds-ratios, though a conventional strategy to facilitate interpretation, is little better. Odds-ratios are not measures of differences in relative likelihood (or relative risk), but of relative odds, which are themselves non-linear transformations of differences in likelihood, dependent on the (unobserved) probability of the outcome in the reference category and equally problematic to interpret. A simple solution becoming common in the social and health sciences is to transform coefficient estimates directly to marginal or discrete change in probability of the outcome. Simply put, a one unit difference in independent variable x can be interpreted as associated with a difference of b in the probability of observing the outcome y, in the same way model estimates are interpreted in the linear regression, conditional on stated levels of other covariates in the model.

5 The “don’t know” responses were predominantly among the home group (42) compared to the facility group (3).

6 There was concern that linguistically and culturally there is little difference between “very good” and “good,” which is why they are coded together and not separated.

7 This figure is according to the Guatemala *Encuesta Nacional de Salud Materno Infantil* 2008 (ENSMI-2008/09), which was the latest available at the time of analysis.

8 Only 16 women (from both groups) intended to give birth in a health facility, but would not recommend it to others. Explanations for their unwillingness to recommend centered on feeling uncomfortable telling others what to do.

9 Women have confidence with their decision to give birth at home because their *comadrona* (traditional midwife) was seen as capable and treated her well/with kindness, was with her at every moment and never left. Some cited the benefit of the *comadrona* being able to speak her language. Many women mentioned having the freedom to ask for food, drink or to use the *temazcal* (traditional sauna) whenever they desired. The minority of the facility birth group who would not recommend a facility birth to someone else cited specific reasons for not liking the care they received, such as being cold, no *temazcal*, no food, not allowing her family in, a lack of privacy, and getting an episiotomy without asking her permission first.
